# Cell- and biological-based drug delivery vectors for cancer treatment

**DOI:** 10.3389/fmolb.2026.1894677

**Published:** 2026-08-06

**Authors:** Maria Zavali, Antonios Georgas, Aggeliki Visvardi Bouga, Angelo Ferraro, Evangelos V. Hristoforou

**Affiliations:** Laboratory of Electronic Sensors, School of Electrical and Computer Engineering, National Technical University of Athens, Athens, Greece

**Keywords:** biological-based, cell-based, doxorubicin, microalgae, targeted drug delivery, vectors for cancer therapy

## Abstract

The introduction of cell- and biological-based (CBb) vectors for drug delivery has paved new pathways in cancer therapy, capitalizing on the distinct properties inherent to various microbial systems. In this review, we take a critical look at a range of promising drug delivery platforms, including polymeric micelles, bacterial outer membrane vesicles (OMVs), liposomes, fungi, yeast, bacteria, red blood cells stem cells, virus and microalgae. Our focus is on evaluating their efficacy, targeting capabilities, and biocompatibility. Key findings suggest that Daptomycin-Doxorubicin (Dap-DOX) micelles enhance cytotoxicity and selectively accumulate in tumors through the enhanced permeability and retention (EPR) effect. Likewise, bacterial-derived magnetosomes appear to offer significant tumor suppression and show reduced cardiac toxicity compared to traditional doxorubicin (DOX) treatments. Additionally, RBC-based systems have demonstrated effective targeting and delivery of DOX to cancer sites, surpassing the performance of free drug formulations. The discussed works mainly focused on the use of DOX, however we also reviewed papers where other chemotherapeutic agents have been used. Among the reviewed platforms, microalgae stand out as particularly promising due to their biocompatibility, poor ability to multiply inside human body, and ability to boost drug loading efficiency, facilitating targeted delivery. Evidence points to microalgae’s potential to enhance the therapeutic index of anticancer drugs, as exemplified by the quick drug release behavior of cell vesicles loaded with doxorubicin in acidic tumor microenvironments. Despite these promising developments, challenges persist, especially in the optimization of microbial engineering and the scalability required for clinical applications. We suggest that microalgae offer a compelling platform for future research and development in cancer drug delivery, highlighting the need for further exploration to unlock their full potential in oncological therapies.

## 1 Introduction

Cancer continues to be a major cause of illness and death globally, presenting formidable challenges in treatment due to its diverse nature and the intricate environments within tumors. Traditional therapies, such as chemotherapy, radiotherapy, and immunotherapy, often encounter obstacles like systemic toxicity, limited bioavailability, and the emergence of drug resistance. These issues highlight an urgent need for innovative drug delivery systems designed to boost therapeutic effectiveness while reducing harmful side effects.

In recent years, advancements in nanotechnology and biotechnology have opened up the potential of microorganism-based drug delivery vectors as a promising alternative to conventional systems. Microorganisms, such as bacteria, fungi, and microalgae, have distinct attributes that can be leveraged for targeted drug delivery. For example, engineered bacterial outer membrane vesicles (OMVs) have exhibited significant cytotoxic effects and tumor growth inhibition in preclinical models, indicating their promise as carriers for chemotherapeutic agents like DOX (Kuerban et al., 2020). Likewise, delivery systems based on red blood cells have shown improved targeting abilities, effectively delivering DOX to cancer lesions while minimizing systemic toxicity (Wang et al., 2021).

Among these various microorganism-based platforms, microalgae stand out as compelling candidates for drug delivery applications. Their natural biocompatibility, capacity to encapsulate therapeutic agents, and potential for targeted delivery make them ideal for enhancing anticancer treatment efficacy. Research suggests that microalgae can increase drug loading efficiency and facilitate the release of therapeutic agents in response to the acidic conditions of the tumor microenvironment, thus optimizing chemotherapy outcomes (Huang et al., 2024). Moreover, microalgae can be engineered to produce oxygen under laser irradiation, which might help alleviate tumor hypoxia and improve the effectiveness of chemotherapeutic agents (Huang et al., 2024).

**FIGURE 1:**
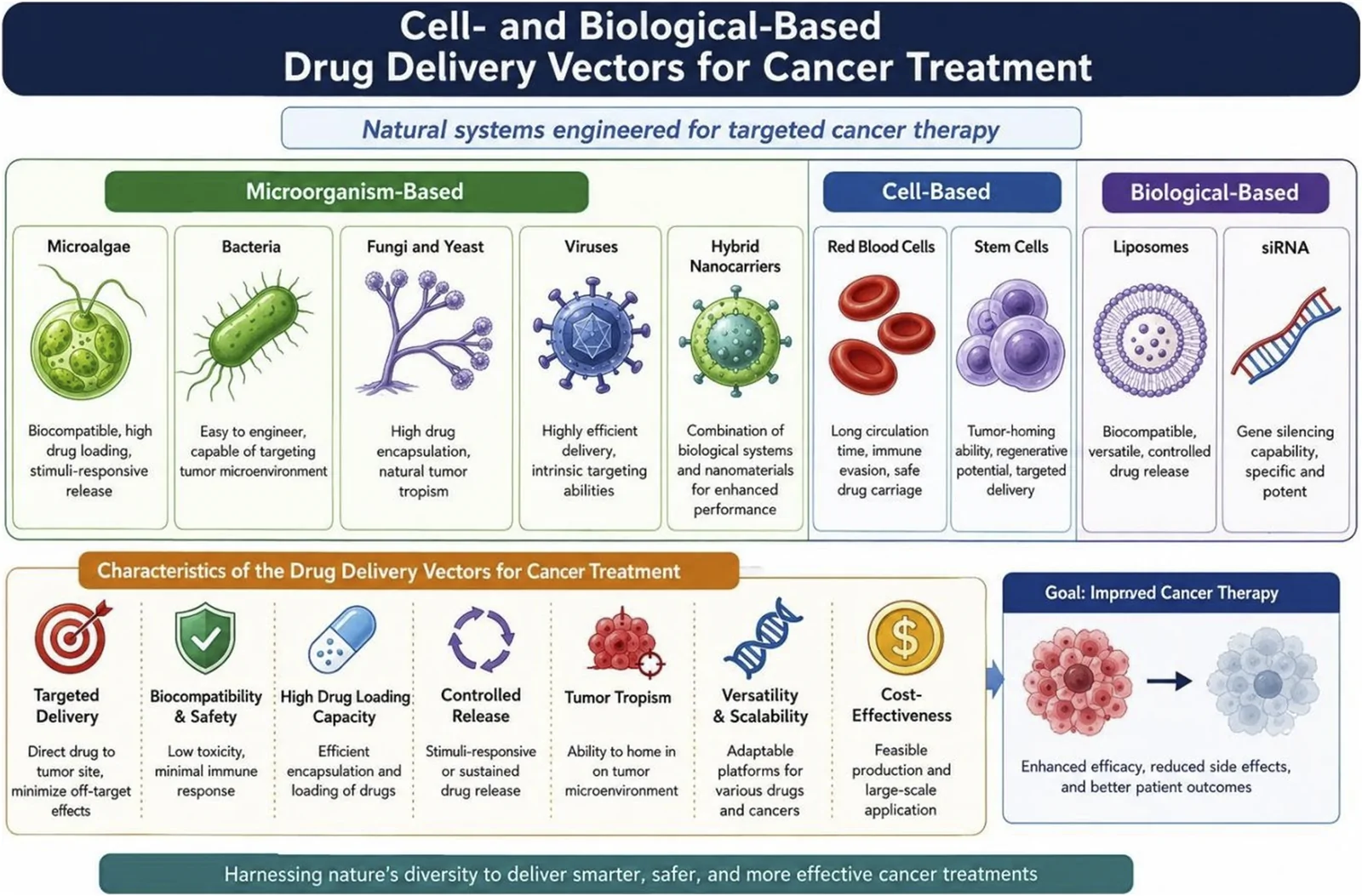
Categories and key characteristics of drug delivery vectors.

This review seeks to provide a comparative overview of various CBb drug delivery systems, as they are presented in Figure 1, with a particular emphasis on microalgae. Although there is an expanding body of evidence supporting the effectiveness of these platforms, it is crucial to recognize the limitations and gaps in current research. Issues such as production scalability, regulatory obstacles, and the potential immunogenicity of engineered microorganisms remain significant areas for further exploration. By underscoring the advantages of microalgae and their potential to address the challenges associated with traditional cancer therapies, this review aims to contribute to the ongoing discussion on innovative drug delivery strategies in oncology.

In this work, we evaluated microalgae-based drug delivery systems for cancer therapy, aiming to explore their potential as an effective platform for enhancing anticancer treatment. Our objective was to investigate the feasibility of using microalgae as biocompatible and safe drug carriers while addressing the challenges associated with targeted drug delivery and biosafety. This evaluation was based on a comparative analysis of microalgae with established drug delivery platforms, classifying them into cell-based and biologically derived delivery systems. Our analysis highlighted several important advantages of microalgae as drug carriers, including their ability to protect encapsulated drugs from degradation under harsh conditions, their satisfactory drug-loading capacity, low cultivation cost, excellent biocompatibility, non-pathogenic nature, and potential to reduce chemotherapy-induced toxicity by minimizing damage to healthy tissues. In addition, microalgae offer opportunities for surface engineering and functional modification (Banis et al., 2022; Savvidou et al., 2022), making them highly versatile platforms for targeted drug delivery. Although microalgae-based drug delivery systems show considerable promise, further research is needed before their clinical application. Future efforts should focus on optimizing carrier design, improving drug loading, and thoroughly evaluating their safety and therapeutic efficacy. Overall, microalgae offer a sustainable and versatile platform for the development of advanced cancer drug delivery systems.

Relevant publications were identified using Google Scholar by applying combinations of the following keyword: “nanocarriers”, “nanoparticles”, “microalgae”, “cell vectors”, “biological vectors”, “doxorubicin” and “drug delivery”. The search focused primarily on biotechnology articles published within the last 5–10 years to ensure that recent advances were included. Articles were selected based on their relevance to the topic, scientific quality and results, contribution to the proof of concept that microalgae-based nanocarriers for drug delivery can be highly competitive towards cell and biological vector.

## 2 Comparison framework

For this review, drug delivery carriers are classified into two main categories: cell-based and biological-based delivery vectors. Cell-based delivery systems consist of living cells combined with synthetic therapeutic cargo, such as biotechnologically engineered red blood cells or stem cells loaded with nanoparticles and/or anticancer drugs. In contrast, biological-based delivery systems integrate biological materials or living organisms with synthetic components, or *vice versa*. These platforms include combinations of microalgae, bacteria, viruses, mRNA, siRNA, fungi, yeast, micelles with liposomes, polymeric nanoparticles, metallic nanoparticles, and anticancer agents. Throughout the literature, these platforms are commonly described as biohybrid drug delivery systems, synthetic delivery systems, biohybrid microrobots, or drug-loaded microrobots, reflecting their innovative bioengineered design and multifunctional therapeutic capabilities.

**FIGURE 2:**
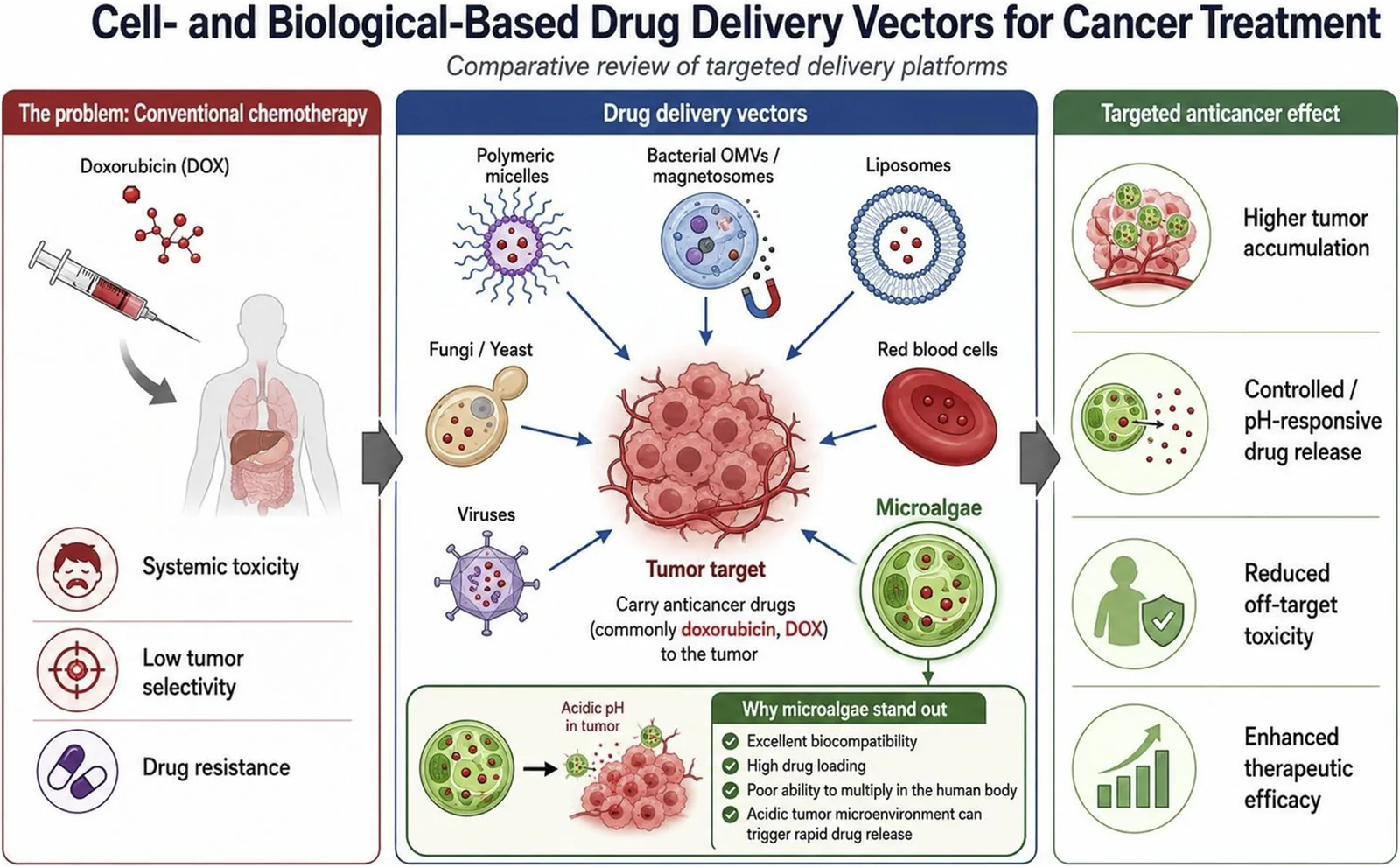
Comparison of targeted drug delivery vectors highlighting the relative advantages of microalgae-based systems versus conventional delivery platforms.

When assessing CBb drug delivery vectors for cancer therapies, we established several criteria to ensure a thorough comparison of their effectiveness, safety, and overall potential. The primary parameters considered were tumor targeting efficiency, cytotoxicity, biocompatibility, drug loading capacity, release profiles, and safety profiles Figure 2.

Tumor Targeting Efficiency. An effective drug delivery system must preferentially accumulate at tumor sites to enhance therapeutic outcomes while reducing systemic side effects. Dap-DOX micelles, for example, achieve selective tumor site accumulation through the enhanced permeability and retention (EPR) effect, proving to be more cytotoxic than free DOX (Guo et al., 2021). Similarly, red blood cell (RBC)-based systems have shown effective targeting of cancer cells, highlighting their potential for precise drug delivery (Wang et al., 2021).

Cytotoxicity and Efficacy. We evaluated the cytotoxic effects of various delivery systems using both *in vitro* and *in vivo* studies. Notably, doxorubicin-glucose conjugated RBCs (Dox-gluRDVs) demonstrated superior anticancer activity compared to free DOX, suggesting enhanced therapeutic efficacy (Wang et al., 2021). Additionally, bacterial OMVs showed significant tumor growth inhibition in animal models, indicating their promise as effective drug carriers (Kuerban et al., 2020).

Safety Profiles. Ensuring safety is crucial in developing drug delivery systems. Drug-loaded bacterial magnetosomes (DBMs) were associated with significantly reduced cardiac toxicity compared to conventional DOX treatment, suggesting a promising safety profile (Sun et al., 2007). However, while micelles and RBCs exhibited favorable biocompatibility, we must further investigate their long-term safety implications.

Drug Loading Capacity and Release Profiles: Effective drug delivery systems must load and release therapeutic agents efficiently. Polymeric micelles achieved a DOX-loading content of 33.64% with a sustained release profile, indicating their potential for prolonged therapeutic action (Guo et al., 2021). Moreover, microalgae-based systems showed enhanced drug loading efficiency and rapid release in acidic tumor microenvironments, which may improve chemotherapy effectiveness (Huang et al., 2024).

Limitations and Gaps in Evidence. Despite these promising findings, several limitations must be considered. Many studies occur *in vitro* or in animal models, which may not fully mirror human physiological conditions. Furthermore, the diversity in methodologies and assessment techniques across studies complicates direct comparisons. There is an ongoing need for long-term safety and efficacy data, particularly regarding potential immunogenic responses and the biodistribution of these vectors in clinical settings.

In summary, while current evidence suggests that microorganism-based drug delivery vectors, especially microalgae, hold significant promises for cancer treatment, further research is necessary to address existing gaps and validate these systems in clinical applications. The innovative potential of microorganisms in drug delivery underscores the importance of continued exploration in this evolving field.

Table 1 provides a comparative overview of various drug delivery platforms, focusing on their tumor targeting capabilities, payload capacity, biocompatibility, evidence level, and primary advantages. Each platform is assessed based on qualitative descriptors derived from the literature, with specific citations for each claim. This structured comparison allows for a clear understanding of the strengths and limitations of each platform in the context of drug delivery systems.

**TABLE 1:** Comparative analysis of drug delivery platforms.

Platform	Tumor targeting	Payload capacity	Biocompatibility	Evidence level	Primary advantages
Microalgae	Effective targeting (Zhang et al., 2024)	High capacity (Zhang et al., 2024)	Excellent (Huang et al., 2024)	*In vivo* (An et al., 2024)	Enhanced drug loading and targeted delivery (Huang et al., 2024)
Bacteria	Effective targeting (Huang et al., 2022)	High capacity (Kuerban et al., 2020)	Good (Huang et al., 2022)	*In vivo* (Kuerban et al., 2020)	Active mobility and tumor targeting (Huang et al., 2022)
Liposomes	Enhanced targeting (Lim et al., 2023)	Moderate capacity (Bishoyi et al., 2025)	Good (Aloss and Hamar, 2023)	*In vivo* (Lim et al., 2023)	Improved safety profile (Aloss and Hamar, 2023)
Red Blood Cells	Effective targeting (Wang et al., 2021)	Moderate capacity (Wu et al., 2021)	Excellent (Wang et al., 2021)	*In vivo* (Wang et al., 2021)	Simultaneous therapeutic and diagnostic delivery (Wu et al., 2015)
Viruses	Effective targeting (Bruno et al., 2025)	Moderate capacity (Bruno et al., 2025)	Variable (Bruno et al., 2025)	Review (Bruno et al., 2025)	Potential standard therapy (Bruno et al., 2025)
Fungi	Limited evidence suggests effective targeting (Huang et al., 2022)	Limited capacity (Huang et al., 2022)	Limited evidence suggests good (Huang et al., 2022)	Review (Huang et al., 2022)	Innovative solutions (Pandey and Pandey, 2024)
Bacterial Outer Membrane Vesicles (OMVs)	Effective targeting (Pandey and Pandey, 2024)	High capacity (Kuerban et al., 2020)	Good (Kuerban et al., 2020)	*In vivo* (Kuerban et al., 2020)	Active participants in cancer therapy (Kuerban et al., 2020)

Key findings from the table highlight that microalgae-based systems exhibit several characteristics. They demonstrate effective tumor targeting and high payload capacity, supported by *in vivo* evidence (Zhang et al., 2024). Additionally, microalgae are noted for their excellent biocompatibility and ability to enhance drug loading and targeted delivery (Huang et al., 2024). These attributes make microalgae a promising candidate for drug delivery applications.

However, the evidence supporting these claims is not without limitations. While microalgae show promise, the majority of studies are at the *in vivo* level, with limited clinical data available. This gap highlights the need for further research to validate these findings in clinical settings. Additionally, while microalgae are presented as strong candidates, the comparative advantages over other platforms, such as bacteria and liposomes, require more comprehensive studies to establish superiority.

## 3 Cell-based drug delivery vectors

In the realm of drug delivery, cell-based systems (CB-DDSs) capitalize on the natural biological characteristics of various cell types to improve drug targeting and bioavailability (Sergazy et al., 2025), particularly for cancer therapies. RBCs and stem cells stand out as leading candidates in this area, each bringing distinct benefits and challenges to the table when it comes to delivering therapeutic agents.

### 3.1 Red blood cells

RBCs have gained attention as a promising drug delivery platform due to their biocompatibility, extended circulation time, and adeptness at navigating the body’s vascular system. Recent research indicates that DOX can be effectively loaded into RBCs, with a loading capacity of about 0.84 ± 0.09 mg/mL. This RBC-based delivery system appears capable of efficiently targeting cancer cells, allowing for the direct delivery of DOX to tumor sites (Wang et al., 2021). Moreover, formulations like Dox-gluRDVs have shown enhanced anticancer activity compared to free DOX, both *in vitro* and *in vivo*, suggesting a potential for greater therapeutic efficacy through targeted delivery approaches (Wu et al., 2021). The ability of RBCs to encapsulate therapeutic agents without impairing their natural functions, such as movement in micromotor applications, further highlights their versatility as drug carriers (Wu et al., 2015).

### 3.2 Stem cells

Turning to stem cells, particularly mesenchymal stem cells (MSCs), these cells have drawn interest in their unique capabilities, such as homing to tumor sites and releasing therapeutic agents in a controlled fashion. Extracellular vesicles (EVs) derived from MSCs show substantial promise for drug delivery. Studies suggest that EVs can be isolated from both MSC-conditioned media and other sources like milk, albeit with varying yields (Mukhopadhya et al., 2023). The intrinsic ability of stem cells to target tumor microenvironments offers an attractive strategy for focused therapy. However, we must address challenges related to optimizing the loading and release profiles of therapeutic agents from these cells. In addition, xenogeneic MSCs may escape growth-control pathways and become cancer cells.

Mesenchymal stem cells (MSCs) are highly promising vehicles for targeted cancer therapy because of their natural tropism (migration ability) toward the hypoxic, inflammatory microenvironments of tumors. By combining MSCs with nanotechnology, researchers can load these cells with potent therapeutic payloads, effectively evading immune clearance while precisely delivering drugs to metastatic sites following intravenous administration (Li et al., 2015). Through genetic engineering, MSCs can be modified to selectively localize within tumors and release therapeutic molecules, including interferons (IFN-β and IFN-γ), interleukins (IL-12 and IL-24), tumor necrosis factor-related apoptosis-inducing ligand (TRAIL), or suicide gene/prodrug systems. Furthermore, MSCs have been explored as vehicles for the delivery of oncolytic viruses and nanoparticle-based drug formulations. Incorporation of nanoparticles into MSCs has been shown to suppress tumor growth both *in vitro* and in rodent cancer models *in vivo* (Khonakdar-Tarsi et al., 2017). In particular, covalent attachment of nanoparticles to the MSC surface enhances drug-loading capacity and improves delivery to tumor sites. Various nanoparticle-based anti-angiogenic platforms, including gold, silica, silicate, diamond, silver, and copper nanoparticles, have demonstrated inhibitory effects on tumor growth. In addition, glycolic acid polyconjugates have been used to improve nanoparticle delivery in human MSCs, while fluorescent nanoparticles, such as coumarin-6, enable non-invasive tracking of MSC migration and intratumoral localization. Despite these promising findings, further optimization of nanoparticle biosafety, long-term stability, therapeutic efficacy, and controlled drug release is required before clinical translation.

Despite the exciting potential of RBCs and stem cells as drug delivery vectors, several limitations and knowledge gaps remain. While RBCs show effective drug targeting, their long-term safety and potential immunogenicity in clinical settings require more thorough investigation. Similarly, although stem cells have favorable targeting properties, the variability in EV yield and composition presents obstacles for standardization and reproducibility in therapeutic applications.

## 4 Biological-based drug delivery vectors

### 4.1 Bacteria as drug delivery vectors in cancer treatment

In recent years, bacteria-based DDS have gained recognition as promising tools for targeted cancer therapy. These systems capitalize on the unique properties of bacteria, which can inherently target tumors and enhance therapeutic effectiveness. This section explores three main bacterial delivery methods: attenuated bacteria, bacterial OMVs, and magnetosomes. We also delve into their safety profiles and potential drawbacks.

#### 4.1.1 Attenuated bacteria

Attenuated bacterial strains, such as *Salmonella* sp. and *Listeria* sp., have shown potential in selectively targeting and proliferating within tumor microenvironments. These bacteria can be genetically modified to deliver therapeutic agents directly to cancer cells, which may amplify the localized treatment effect while reducing systemic toxicity. For instance, engineered *Salmonella* has demonstrated notable tumor suppression in preclinical studies, indicating a promising therapeutic index when compared to traditional chemotherapy (Huang et al., 2022). Nevertheless, the use of live attenuated bacteria poses safety concerns, particularly the risk of infection and unintended immune responses in patients.

#### 4.1.2 Bacterial outer membrane vesicles (OMVs)

OMVs have emerged as versatile drug delivery vehicles due to their natural capability to encapsulate and transport bioactive molecules. Recent research has highlighted that OMVs loaded with DOX-OMVs show significant cytotoxicity against cancer cell lines and inhibit tumor growth *in vivo*, particularly in models like A549 tumor-bearing BALB/c nude mice (Kuerban et al., 2020). The possibility of engineering OMVs to express specific ligands or peptides enhances their targeting precision, allowing for accurate delivery to malignant tissues (Pandey and Pandey, 2024). However, the immunogenicity and potential for off-target effects of OMVs require comprehensive evaluation to ensure they are safe for patients.

Outer membrane vesicles (OMVs) are a subtype of extracellular vesicles (EVs) that are naturally released by Gram-negative bacteria. These nanosized proteoliposomes contain outer membrane lipids, proteins, enzymes, bacterial nucleic acids, and other bioactive molecules, making them attractive nanocarriers for drug delivery. Their drug-loading capacity (LC%) represents the mass percentage of the final formulation that consists of the therapeutic agent. In layer-by-layer or multi-layered engineered OMV systems, drug-loading capacity is defined as the amount of drug encapsulated within the vesicle and its surface layers relative to the total carrier weight. Attenuated *Klebsiella* pneumoniae-derived OMVs have been shown to efficiently deliver doxorubicin (DOX) to A549 cells, resulting in enhanced antitumor activity against non-small-cell lung cancer compared with free DOX, without significant *in vivo* toxicity. In addition, the intrinsic immunogenicity of OMVs promoted macrophage recruitment within the tumor microenvironment, further enhancing the therapeutic efficacy of the encapsulated drug (Kuerban et al., 2020). A549 cells were cultured overnight and then treated with OMVs, DOX (20 μg/mL), 20 μg/mL of DOX loaded in OMVs (DOX-OMV) or loaded in liposome (DOX-LIPO) separately for 12 h, or incubated with DOX-OMV for 0, 6, 12 and 24 h separately.

It is hypothesized that microalgae as a biological-based drug delivery carrier possess a higher drug-loading capacity than conventional nanoscale drug delivery vectors due to their substantially larger size. While most used drug carriers, such as liposomes, polymeric nanoparticles, and inorganic nanoparticles, typically have dimensions in the nanometer range, microalgae are generally several micrometers in size. This larger physical volume provides a significantly greater internal and surface area for the encapsulation or adsorption of therapeutic agents, thereby increasing their potential drug-loading capacity. Consequently, microalgae may serve as highly efficient drug carriers capable of accommodating larger quantities of therapeutic cargo compared with conventional nanocarriers.

#### 4.1.3 Magnetosomes

Magnetosomes, which are membrane-bound magnetic nanoparticles produced by certain bacteria, present an innovative strategy for targeted drug delivery (Alphandéry, 2014). These structures can be loaded with chemotherapeutic agents, such as DOX, and directed to tumor sites using external magnetic fields. Research suggests that doxorubicin-loaded bacterial magnetosomes (DBMs) exhibit significant cytotoxicity against various cancer cell lines while remaining stable in serum over extended periods. Notably, DBMs have demonstrated reduced cardiac toxicity compared to conventional doxorubicin formulations, underscoring their potential for safer cancer treatments (Sun et al., 2007; 2008). Nonetheless, challenges like the scalability of magnetosome production and the necessity for strict quality control must be tackled before these can be used clinically.

### 4.2 Fungi and yeast-based drug delivery systems

Fungi and yeast have emerged as compelling candidates for drug delivery systems in cancer therapy. Their natural targeting properties, substantial payload capacities, and the potential for genetic engineering make them particularly promising for enhancing therapeutic effectiveness. These microorganisms exhibit unique biological characteristics that can be leveraged to improve the precision and efficacy of cancer treatments.

One of the standout features of fungi and yeast as drug delivery vectors lies in their inherent ability to target diseased tissues. By engineering these microorganisms to express specific ligands or peptides, we can facilitate precise targeting of cancer cells. This approach seems to enhance the therapeutic index while minimizing off-target effects (Pandey and Pandey, 2024). Such targeted therapy is especially relevant in cancer treatment, where conventional methods often suffer from systemic toxicity and limited efficacy. Engineered microorganisms offer the potential to boost anti-cancer efficacy and address the challenges posed by traditional therapies, including drug resistance and adverse side effects (Huang et al., 2022).

The payload capacity of fungi and yeast further underscores their potential in drug delivery. For example, small yeast-derived nanoparticles (YCW NPs) have shown greater efficiency in controlling tumor growth than larger particles, indicating that size and formulation profoundly impact therapeutic outcomes. Moreover, YCW NPs have been found to alter the immunosuppressive microenvironment within tumors and nearby lymph nodes, which is crucial for boosting anti-tumor immunity (Xu et al., 2022). This dual capability to deliver therapeutic agents while also stimulating an immune response against the tumor underscores their potential benefits.

However, despite these promising features, there are still limitations and gaps in our current understanding of fungi and yeast-based delivery systems. The engineering of these microorganisms for specific targeting and payload delivery remains in the early stages, and further research is essential to fully elucidate their mechanisms of action and optimize their therapeutic potential. Additionally, it is crucial to rigorously evaluate the safety profiles of these engineered systems to ensure they do not provoke unintended immune responses or other adverse effects in patients.

In the broader context of drug delivery vectors, fungi and yeast offer a compelling avenue for exploration, particularly when compared to other candidates like microalgae, which are also being considered for drug delivery applications (Huang et al., 2022). While microalgae possess unique properties that enhance their utility as drug delivery vectors, the advantages of fungi and yeast, such as their ability to be engineered for targeted therapy and their capacity to modulate the tumor microenvironment, merit further investigation.

Beyond targeting improvements, cell-based DDS often display favorable pharmacokinetic properties. For instance, polymeric micelles have been noted for their desirable DOX-loading content of 33.64% and an encapsulation efficiency of 48.90%, coupled with a sustained release profile conducive to prolonged therapeutic action (Mahani et al., 2023). Such sustained release is particularly advantageous in the acidic tumor microenvironment (TME), where rapid drug release enhances chemotherapy efficiency (Huang et al., 2024).

Moreover, microbial vectors like bacterial OMVs have shown significant anticancer activity, with DOX-OMVs producing notable cytotoxic effects and substantial tumor growth inhibition *in vivo* (Kuerban et al., 2020). This underscores the potential of microorganisms not just as passive carriers but also as active participants in cancer therapy.

However, while these advantages are compelling, it is crucial to recognize the limitations and research gaps that persist. The scalability of microorganism-based DDS and the regulatory hurdles for their clinical application pose significant challenges. Even though current evidence supports their efficacy, further research is necessary to fully understand their long-term safety and potential immune responses.

In summary, microorganism-based drug delivery vectors offer a promising alternative to traditional approaches, with their capability to enhance drug targeting, improve pharmacokinetics, and reduce systemic toxicity. As research progresses, especially regarding microalgae and their biocompatibility, the potential for these innovative systems to enhance cancer treatment paradigms becomes ever more evident. Continued exploration and refinement of these microbial vectors may pave the way for more effective and safer therapeutic strategies in oncology.

### 4.3 Microalgae as drug delivery vectors in cancer treatment

**FIGURE 3:**
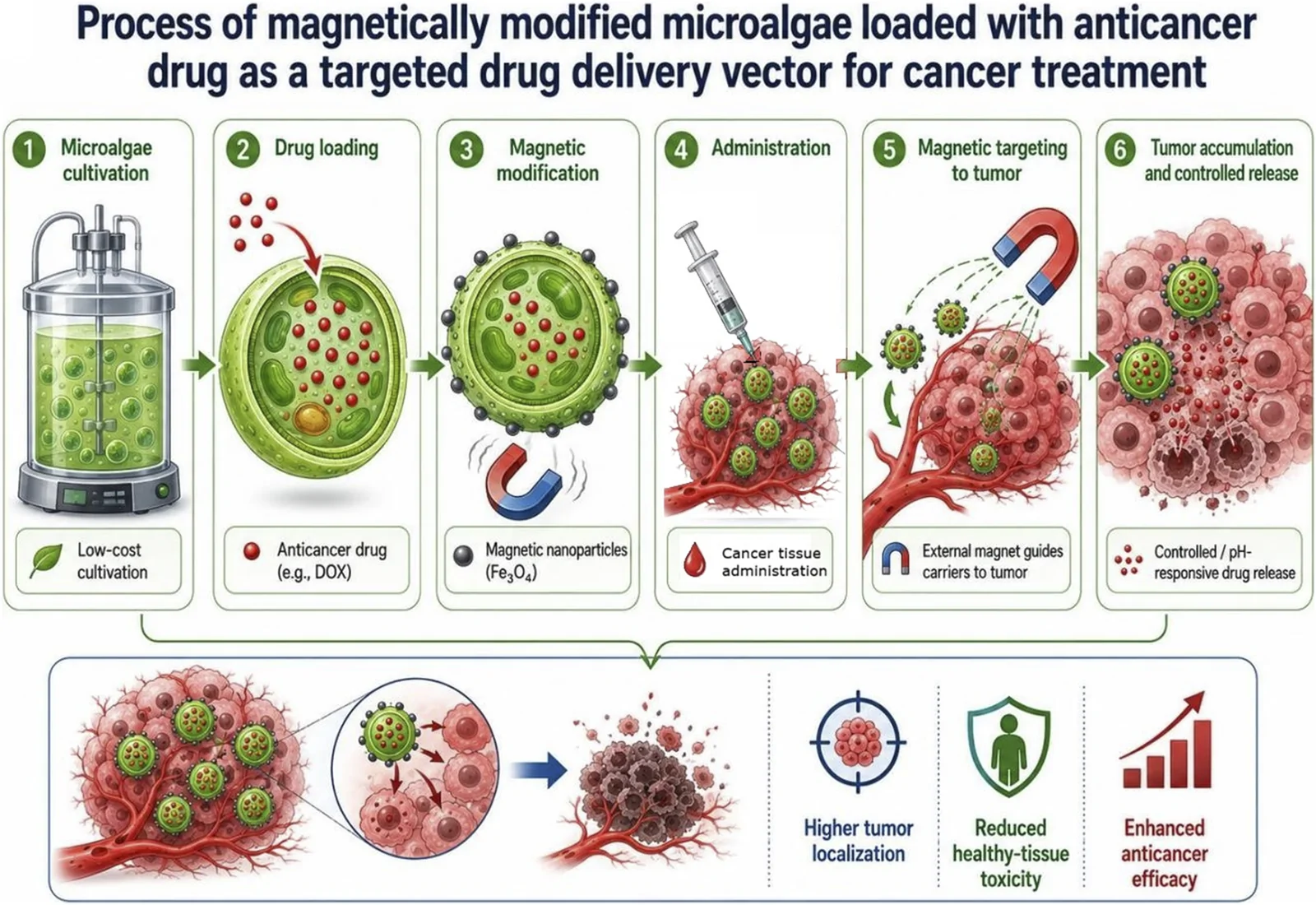
Targeted drug delivery process for cancer therapy using microalgae as a vector.

Microalgae have gained attention as promising drug delivery vehicles, particularly for cancer therapy. Figure 3 is a schematic representation of the targeted drug delivery process using magnetically modified microalgae as a vector. Their distinctive features—such as photosynthesis, oxygen production, and inherent biocompatibility—make them innovative vectors for the targeted delivery of anticancer agents. These organisms have the potential to improve drug loading efficiency and facilitate targeted delivery, which could help overcome some of the limitations of conventional chemotherapy (Huang et al., 2024).

One remarkable advantage of microalgae is their ability to produce oxygen through photosynthesis. In the often hypoxic tumor microenvironment (TME), this trait can be especially beneficial. For example, research has shown that microalgae-based systems like CV@DOX—nanoscale or micro-robotic carriers that pair microalgae (such as Chlorella vulgaris) with drugs like doxorubicin—can rapidly release drugs in the acidic conditions typical of the TME, potentially enhancing chemotherapy efficacy. Moreover, when exposed to specific laser irradiation at 650 nm, these systems can generate oxygen, which may help alleviate tumor hypoxia and improve therapeutic outcomes (Liang et al., 2024).

Furthermore, current studies have explored the potential of microalgae in creating biohybrid microrobots. These microrobots harness the natural propulsion and phototaxis of microalgae for efficient cargo delivery (Zhang et al., 2024). Engineered to enhance the anticancer properties of delivered agents while reducing systemic toxicity, these biohybrid systems address a common challenge faced in traditional cancer therapies (Huang et al., 2022).

Additionally, microalgae have been used to produce extracellular vesicles known as nanoalgosomes, which are efficiently taken up by mammalian cell lines (Adamo et al., 2021). This feature highlights their potential as a renewable and sustainable source for biopharmaceutical production, further expanding their applicability in drug delivery systems (Bolaños-Martínez et al., 2022).

Extracellular vesicles (EVs) are membrane-bound vesicles released by cells, with a lipid bilayer membrane on the outside, which are widely found in various body fluids and contain biological macromolecules such as DNA, RNA, lipids and proteins on the inside. The advantageous properties of EVs include composition specificity, specific targeting, circulatory stability, active penetration of biological barriers, high efficiency as drug delivery vehicles and anticancer vaccines, oxidative phosphorylation activity, enzymatic activity facilitate the clinical application of EVs (Zeng C. et al., 2022). Extracellular vesicles (EVs) present high stability within biological fluids (e.g., blood, urine, cerebrospinal fluid, saliva) (Boukouris and Mathivanan, 2015). Their lipid bilayer shields internal cargo (RNA, proteins, lipids) from enzymatic degradation. Moreover, membrane-associated proteins contribute to prolonged circulation by reducing immune recognition and clearance However, isolation procedures and repeated freeze–thaw cycles diminish their stability.

The promising use of microalgae in drug delivery is further supported by their unique biological properties. These organisms have a natural tendency to accumulate and selectively release therapeutic agents at tumor sites, taking advantage of the enhanced permeability and retention (EPR) effect, a well-documented phenomenon in various drug delivery systems (Guo et al., 2021). This selectivity is crucial for minimizing systemic toxicity and maximizing the therapeutic index of anticancer drugs.

**FIGURE 4:**
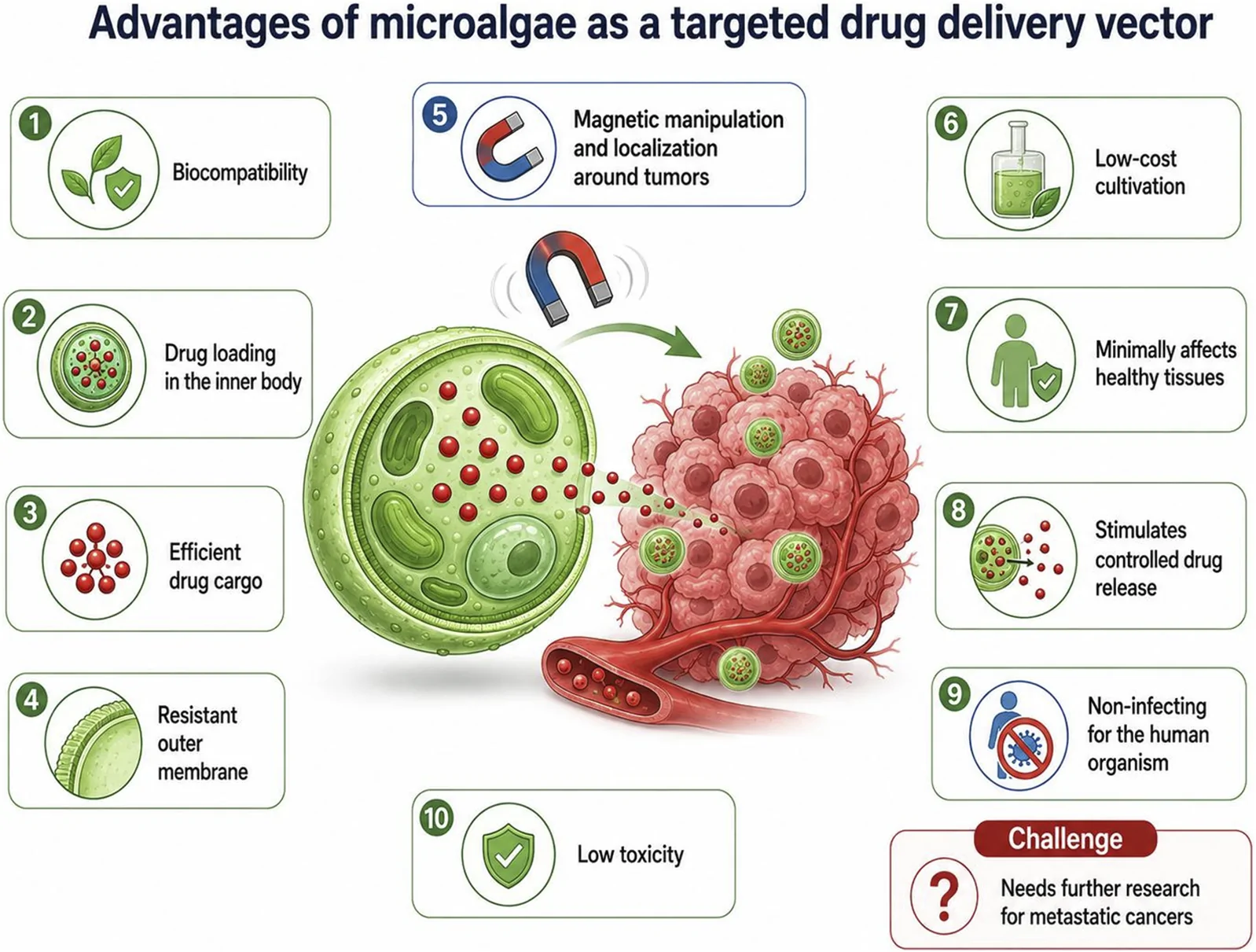
Schematic overview of the advantages of microalgae as a targeted drug delivery vector.

Moreover, although some studies hint at the potential of microalgae to enhance drug loading efficiency, there is a lack of comparative analyses with established drug delivery systems, such as polymeric micelles or red blood cell-based carriers. For instance, polymeric micelles are known for their favorable drug loading content and sustained release profiles, which are critical for effective cancer treatment (Mahani et al., 2023). Similarly, red blood cell-based systems have shown effective targeting and efficient drug delivery to cancer cells (Wu et al., 2021). Thus, a thorough comparison of microalgae with these established systems is necessary to confirm their potential advantages as drug delivery vectors Figure 4.

However, despite these encouraging advancements, several challenges and knowledge gaps persist in the field of microalgae-based drug delivery. Key areas requiring further exploration include the scalability of production and the consistency of drug loading efficiency across various microalgal species. While the biocompatibility of microalgae is well-established, the long-term effects of their use in human subjects still need comprehensive evaluation.

In conclusion, while microalgae offer a promising avenue for drug delivery in cancer treatment, additional research is needed to address the existing limitations and fully explore their potential. Integrating microalgae into the broader scope of microorganism-based DDS could provide innovative solutions to challenges faced by traditional systems. However, rigorous studies are essential to substantiate the claims regarding their efficacy and practicality in clinical settings.

#### 4.3.1 Microalgae multiplication rate

Microalgae generally exhibit rapid multiplication rates, with growth kinetics varying according to species, environmental conditions, and cultivation strategies. Under optimal conditions, certain microalgal species can achieve doubling times ranging from several hours to a few days, resulting in high biomass productivity relative to many conventional biological systems. Chlorella vulgaris, Spirulina platensis, and Dunaliella salina are known for their relatively rapid growth and high biomass productivity under optimized culture conditions. Their doubling times generally range from approximately 24–48 h for Chlorella vulgaris, 2–5 days for Spirulina platensis (Kumar et al., 2011), and 1–3 days for Dunaliella salina. However, these growth rates are highly dependent on key environmental parameters, including light intensity, nutrient availability, carbon dioxide (CO_2_) supply, temperature, pH, salinity. Careful optimization of these factors is essential to ensure efficient and scalable microalgal production. Under *in vitro* cultivation conditions, these parameters can be optimized to support rapid growth and biomass production. However, following *in vivo* administration, microalgae are exposed to a markedly different physiological environment where light is absent and the availability of CO_2_ for photosynthesis is severely limited. Consequently, photosynthetic activity and cell proliferation are expected to be substantially reduced or completely inhibited, depending on the microalgal species and the surrounding tissue microenvironment. This restricted proliferative capacity may represent an important safety advantage for microalgae-based drug delivery systems by minimizing the risk of uncontrolled biomass expansion after administration.

Microalgal physiology in outdoor cultivation significantly affects biomass productivity (Velásquez-Orta et al., 2024). Through photosynthesis, microalgae utilize CO_2_ as well as macro- and micronutrients from wastewater, enabling high growth rates and biomass yields that may exceed those of terrestrial grasslands by up to 50-fold. Productivity in open systems is strongly governed by cellular physiology, which is influenced by culture medium composition (e.g., wastewater type), pH, temperature, growth phase, and operational factors such as mixing and harvesting efficiency. Light availability is the primary driver of photosynthetic activity. Maximum biomass accumulation typically occurs during daylight; however, up to 25% of produced biomass may be lost at night due to respiratory metabolism, depending on daytime irradiance and diurnal temperature variations. In the absence of light, photosynthetic energy conversion is halted, reducing adenosine triphosphate (ATP) and nicotinamide adenine dinucleotide phosphate (NADPH) synthesis required for cell growth, whereas excessive irradiance can induce photodamage to chlorophyll. Based on energy and carbon utilization strategies, microalgae are classified as photoautotrophic, heterotrophic, or mixotrophic. Photoautotrophic rely on sunlight as an energy source and CO_2_ as a carbon source. Heterotrophic utilize organic carbon sources in darkness. Mixotrophic cell growth relies both on CO_2_ and organic carbon.

Although promising results are often achieved at laboratory scale, comparable outcomes are difficult to reproduce in industrial photobioreactors (Hawrot-Paw and Ratomski, 2024). Changes in cultivation conditions during scale-up can influence both microalgal growth and metabolic activity. Biomass productivity of selected strains is also strongly dependent on photobioreactor design and capacity, with a general decline in biomass yield observed as production scale increases, regardless of species. Scale-up similarly affects biochemical composition, particularly lipid content. Higher lipid levels were recorded in small-scale 2.5 L photobioreactors (e.g., 26.3% ± 2.2% in Monoraphidium and 13.9% ± 0.3% in Chlorella vulgaris), whereas industrial-scale systems produced significantly lower lipid contents (7.1% ± 0.6% in Oocystis submarina and 10.2% ± 1.2% in Chlorella fusca). Additionally, increasing photobioreactor volume was associated with reduced biomass quality, including higher ash content.

### 4.4 Hybrid nanocarriers

In the field of drug delivery, particularly for cancer treatment, hybrid nanocarrier systems stand at a promising crossroads. By merging biological components with synthetic materials, these systems aim to capitalize on the strengths of both areas to improve drug delivery effectiveness, enhance targeting accuracy, and achieve better therapeutic results. Microorganisms like bacteria, fungi, and microalgae are gaining attention for their ability to aid in the synthesis and functionalization of nanocarriers, addressing some limitations of traditional drug delivery methods.

Polymeric micelles serve as a notable example, offering significant advancements in drug loading and release. For instance, Dap-DOX micelles have shown greater cytotoxicity than free DOX and have demonstrated selective accumulation at tumor sites via the enhanced permeability and retention (EPR) effect, thereby boosting therapeutic efficacy (Guo et al., 2021). Furthermore, these micelles achieved a favorable DOX-loading content of 33.64% and an encapsulation efficiency of 48.90%, with a sustained release profile at the acidic pH levels characteristic of the tumor microenvironment (TME) (Mahani et al., 2023). Such findings underscore the potential of hybrid systems to refine drug delivery kinetics.

The microbial-assisted synthesis of nanocarriers presents innovative solutions to address issues like biocompatibility and targeted delivery. By engineering microorganisms to express specific ligands or peptides, we can facilitate precise targeting of diseased tissues, potentially enhancing therapeutic outcomes (Pandey and Pandey, 2024). For instance, Dectin-1 DEC-installed-micelles have demonstrated superior antifungal activity *in vitro* compared to their non-DEC counterparts, highlighting the promise of microbial engineering in boosting the functional properties of nanocarriers (Inukai et al., 2024).

Moreover, incorporating microalgae into hybrid nanocarrier systems has shown promise. Microalgae-based carriers, such as CV@DOX, have been observed to release drugs rapidly under acidic TME conditions, which can notably enhance chemotherapy efficiency. Additionally, when exposed to laser irradiation, these carriers can produce oxygen, potentially mitigating tumor hypoxia and further improving chemotherapy effectiveness (Liang et al., 2024). This ability highlights the versatility of microalgae as a platform for developing multifunctional hybrid nanocarriers.

However, despite these exciting developments, the field of hybrid nanocarriers still faces several challenges. Issues such as the scalability of microbial synthesis processes, the stability of hybrid systems in physiological conditions, and the potential immunogenicity of biological components require further exploration. Moreover, the intricate interactions between synthetic and biological components in these hybrid systems demand a more profound understanding to optimize their design and functionality.

Thus, hybrid nanocarriers that integrate the capabilities of microorganisms with synthetic materials hold immense promise for advancing drug delivery in cancer treatment. While the inclusion of microalgae and other microorganisms into these systems could lead to groundbreaking therapeutic strategies, further research is vital to overcome existing hurdles and fully harness their clinical potential.

### 4.5 Liposomes

Liposomes, which are spherical vesicles crafted from lipid bilayers, have become a pivotal tool in drug delivery, especially when it comes to cancer therapy. These versatile carriers can encapsulate both hydrophilic and hydrophobic drugs, thereby enhancing the solubility and stability of therapeutic agents—key factors in mitigating the limitations of conventional drug formulations. The underlying mechanisms of liposome-mediated delivery include passive targeting, thanks to the enhanced permeability and retention (EPR) effect, and active targeting through functionalization with specific ligands or peptides that bind to receptors overexpressed on cancer cells (Pandey and Pandey, 2024).

One of the notable strengths of liposomes is their biocompatibility and favorable safety profile. Several liposomal formulations have garnered FDA approval, such as liposomal doxorubicin (Doxil), which have demonstrated improved pharmacokinetics while reducing the cardiotoxicity associated with non-liposomal versions (Aloss and Hamar, 2023). Moreover, the development of functionalized liposomes, including those sensitive to pH and temperature, has been shown to enhance drug accumulation within tumor tissues, which could potentially heighten therapeutic effectiveness (Mirhadi et al., 2022; Aloss and Hamar, 2023). Additionally, research suggests that extending the PEG-linker chain length in folate-linked liposomal formulations may significantly boost tumor-targeting capabilities and antitumor activity *in vivo* (Lim et al., 2023).

However, liposomes are not without their challenges, which could impede their clinical applications. Stability is a major concern; liposomes are prone to degradation in biological environments, possibly leading to premature drug release and diminished therapeutic effects. While they can enhance delivery to tumors, achieving precise targeting is still a hurdle. The heterogeneous nature of tumor microenvironments and the presence of biological barriers can restrict the effectiveness of liposome-based therapies (Liang et al., 2024).

In the current clinical landscape, liposomes have shown promise, particularly in leukemia treatment, where they have been employed to encapsulate and directly deliver anticancer drugs to leukemic cells (Bishoyi et al., 2025). Nevertheless, their overall efficacy compared to traditional therapies remains under scrutiny. Some studies indicate that while liposomes improve safety profiles, they do not always exhibit superior efficacy (Aloss and Hamar, 2023).

### 4.6 Ribonucleic acid - RNA delivery systems

#### 4.6.1 siRNA

Non-viral delivery systems have become widely utilized for the transport of Small interfering RNA (siRNA) and anticancer agents to tumor tissues. The guide strand of siRNA interacts with the RNA-induced silencing complex (RISC), leading to targeted gene suppression (Xue et al., 2024). Currently, siRNA-based anticancer therapies are still under preclinical and clinical investigation, with no siRNA drug yet approved for cancer treatment, mainly due to several challenges in their development (Yan et al., 2026). These challenges include: (1) Immunogenicity, as siRNA molecules may activate immune responses; (2) Toxicity concerns, including sequence-dependent off-target effects caused by siRNA–gene mismatches and toxicity associated with delivery components; (3) Limited *in vivo* stability, due to rapid degradation by ribonucleases and/or renal elimination, resulting in a short circulation time; and (4) Delivery barriers, as siRNAs lack inherent tissue-specific targeting ability. Furthermore, their high molecular weight and hydrophilic nature prevent efficient membrane penetration, and following cellular internalization, siRNAs can become trapped in lysosomes, limiting their therapeutic activity.

Small interfering RNA (siRNA) has gained attention as a promising tool for cancer therapy, primarily because of its capacity to selectively silence genes implicated in tumor development. Despite its potential, delivering siRNA effectively to specific tissues, and inside tumor cells, continues to pose significant hurdles. Its instability in biological settings and the necessity for precise targeting to minimize unintended effects add to these challenges. Nevertheless, recent strides in utilizing microorganism-based carriers have shown promise in overcoming these obstacles.

Microorganisms such as bacteria, fungi, and yeast present distinct advantages and can be used as a “siRNA factory” as well as vectors for drug delivery. They can be genetically modified to enhance targeting capabilities and improve the pharmacokinetic profile of siRNA. For example, microbial-assisted synthesis has emerged as a promising approach to bypass the limitations of conventional drug delivery systems. This strategy allows for the creation of carriers that can preferentially accumulate at tumor sites, leveraging mechanisms like the enhanced permeability and retention (EPR) effect (Guo et al., 2021). Such selective accumulation is vital for achieving maximum therapeutic impact while minimizing systemic side effects.

Among the various microorganism-based carriers, red blood cells (RBCs) have demonstrated potential in delivering chemotherapeutic drugs like doxorubicin (DOX) and might be adaptable for siRNA transport. RBC-based systems have shown proficiency in targeting cancer cells, hinting that similar approaches could be adapted to enhance siRNA delivery to tumors (Liu W. et al., 2025). Additionally, engineering microorganisms to express specific ligands or peptides can aid in the targeted delivery of siRNA, thus boosting the precision of gene silencing efforts (Pandey and Pandey, 2024).

However, despite these advancements, several challenges remain in the realm of siRNA delivery using microorganism-based carriers. The stability of siRNA within these carriers and their efficiency in cellular uptake are critical areas needing further exploration. Moreover, the potential immunogenicity of microbial vectors and their long-term safety in clinical settings must be comprehensively assessed.

While numerous studies have shown the efficacy of microorganism-based carriers in delivering chemotherapeutic agents, research focusing explicitly on siRNA delivery remains limited. This gap underscores the necessity for more focused investigations to refine the design and enhance the functionality of these carriers for gene silencing purposes.

In summary, microorganism-based carriers offer an innovative and potentially effective method for siRNA delivery in cancer therapy. Their capacity to be engineered (overexpression of selected siRNA) for improved targeting and reduced toxicity makes them promising candidates in the search for effective gene therapy solutions. Future research should aim to address current limitations and explore the full potential of these biological systems in the context of siRNA delivery, particularly within cancer therapeutics.

#### 4.6.2 mRNA

Messenger RNA (mRNA) mRNA cancer therapies use protective nanocarriers to shield mRNA from enzymatic degradation and deliver it into target cells. Once inside, the mRNA directs the production of tumor-specific proteins, eliciting a localized anti-tumor immune response. Among available delivery systems, lipid nanoparticles (LNPs) are currently the most clinically validated vector for mRNA delivery (He et al., 2025; Hou et al., 2021). Hajj et al. (2020), demonstrated that lipid nanoparticles (LNPs) enable multiplexed mRNA delivery and efficient *in vivo* gene editing, highlighting their potential as versatile platforms for nucleic acid-based cancer therapies. Gilleron et al. (2013), used image-based analysis to investigate lipid nanoparticle (LNP)-mediated siRNA delivery, intracellular trafficking, and endosomal escape, elucidating key processes involved in intracellular siRNA delivery. Zeng Y. et al. (2022), investigated the delivery barriers and administration routes associated with lipid nanoparticle (LNP)-mRNA formulations, highlighting key challenges affecting their therapeutic application. Messenger RNA (mRNA) has become an emerging class of therapeutic molecules with potential applications in the prevention and treatment of various diseases. For effective *in vivo* activity, mRNA requires reliable and stable delivery platforms that prevent degradation, facilitate cellular internalization, and enable efficient release. Lipid nanoparticles (LNPs) have demonstrated successful clinical translation as mRNA delivery systems, with LNP–mRNA vaccines against coronavirus disease 2019 (COVID-19) representing a significant milestone in the advancement of mRNA-based therapeutics (Hou et al., 2021).

### 4.7 Viruses

Small Viral vectors have gained attention as a promising tool within microorganism-based drug delivery systems, particularly for cancer treatment. These vectors capitalize on the inherent ability of viruses to invade host cells and deliver therapeutic agents—including genes, siRNA, proteins, and small molecules—directly into tumor cells. The actions of viral vectors are complex, incorporating direct oncolytic activity, immune system modulation, and precise gene delivery. Oncolytic viruses, known for selectively targeting and destroying cancer cells while sparing normal tissues, have attracted significant interest because they serve both as therapeutic agents and delivery vehicles, for example, altering viral surface proteins can increase their affinity for particular tumor markers (Mistarz et al., 2021; Bruno et al., 2025).

Clinically, the use of viral vectors in cancer therapy has made notable strides. For instance, talimogene laherparepvec (T-VEC) has received regulatory approval for treating melanoma. Such therapies have shown potential in provoking systemic anti-tumor immunity, which may enhance the effectiveness of concurrent immunotherapies (Huang et al., 2022). Nevertheless, despite these promising outcomes, the field remains in its early stages. Further research is essential to refine vector design, dosing strategies, and patient selection criteria to maximize therapeutic gains.

One of the standout benefits of viral vectors in gene delivery is their high transduction/transfection efficiency and specificity for tumor cells. This specificity often stems from the natural affinity of certain viruses for cancerous tissues, which can be further fine-tuned through genetic engineering. For example, altering viral surface proteins can increase their affinity for particular tumor markers, thereby enhancing targeting accuracy and minimizing off-target effects. Virus-like nanoparticles can also be functionalized through add-and-display strategies to immobilize anticancer drugs for controlled delivery (Biabanikhankahdani et al., 2017). Thermally responsive virus-like particles have further been explored as stimuli-responsive platforms for targeted cancer-drug delivery (Thong et al., 2019). Moreover, the ability of viruses to replicate within tumor cells can amplify therapeutic effects, potentially leading to prolonged anti-cancer responses.

However, the use of viral vectors is not without its challenges. Safety and immunogenicity remain critical concerns, as introducing viral components can trigger strong immune responses, potentially limiting the therapy’s effectiveness or causing adverse effects. Additionally, pre-existing immunity in patients, resulting from prior exposure to viral pathogens, can impede the success of viral vector therapies (Huang et al., 2022). These challenges underscore the importance of carefully weighing risks and benefits in clinical applications.

In the broader landscape of microorganism-based drug delivery, microalgae present a compelling alternative to viral vectors. Their natural advantages, such as biocompatibility, low immunogenicity, and the potential for engineering targeted delivery, make them promising candidates for enhancing cancer treatment strategies (Walker et al., 2019; Zhang et al., 2019; Schmidt et al., 2020; Bishoyi et al., 2025; Bruno et al., 2025). While viral vectors have paved the way for innovative therapies, exploring microalgae as delivery vehicles may offer unique benefits that deserve further study.

Viral and oncolytic vector systems represent significant advancements in cancer therapy, with the potential to transform treatment paradigms. However, continued research is crucial to address the limitations and safety concerns of these vectors while also investigating alternative systems like microalgae, which could complement or enhance their effectiveness in targeted cancer therapy.

### 4.8 Various drug-based formulations

Recent progress for Cisplatin-based liposomal nanocarriers for drug delivery in lung cancer therapy is presented by Mohammad-Jafari et al. (2024). Furthermore theranostic nanoparticles with controlled release of Gemcitabine for MRI and targeted therapy of pancreatic cancer is described by Han et al. (2022). In the research of Papa et al. (2012), Gemcitabine was encapsulated in two types of commonly used nanovectors, namely, poly (lactic-co-glycolic acid) (PLGA) and cholesterol-based liposomes. Kim et al. (2023), presented the use of Doxorubicin, Paclitaxel, Epirubicin, Rapamycin, Thymoquinone in lipid nanocarrier-based drug delivery systems for the therapeutic treatment of lung cancer. Proteins have been explored as drug delivery carriers. The most common proteins used in nanocarrier designs are ferritin, albumin, transferrin, low-density lipoprotein, high-density lipoprotein, and so forth. Albumin-bound paclitaxel is the most well-known available protein-based nanoparticulate delivery system (Ganpisetti et al., 2025). Peptides (such as RGD or F3) are grafted onto nanocarrier surfaces to recognize and bind to specific receptors overexpressed on cancer cells (like integrins or nucleolin) as in breast cancer applications (Omidian et al., 2025). This receptor-mediated endocytosis ensures drugs accumulate only at the tumor site. The transferrin binding peptide (TBP) binds to the transferrin receptor (TfR), which mediates receptor mediated endocytosis, making it a valuable tool for brain-targeted drug delivery. Its application in targeted doxorubicin delivery for colorectal cancer highlights its potential beyond neurological applications. Biohybrid magnetic algae (Coscinodiscus granii) microrobots (algebots) enable targeted, non-contact drug delivery through intelligent navigation, magnetic control, and enhanced tissue penetration, achieving improved doxorubicin delivery and reduced bladder tumor burden without systemic toxicity (Lin et al., 2026). Lipid-based vectors for the co-delivery of siRNA and anticancer drugs to overcome tumor multidrug resistance (MDR) have been described by Xue et al. (2024). Doxorubicin (Dox) is a substrate of the ABC transporters ABCB1, ABCG2, and ABCC1, which play key roles in the development of the MDR phenotype. siRNA-mediated silencing of these transporter genes enhances the sensitivity of breast cancer cells to Dox. Beyond reversing drug resistance, siRNAs can selectively suppress oncogene expression and have also shown potential in cancer immunotherapy by stimulating innate immune responses and modulating the tumor microenvironment (TME). The review of He et al. (2025), aims to explore how intratumoral delivery of mRNA-loaded in lipid nanoparticles (LNPs) enhances tumor immunotherapy. LNPs protect mRNA from enzymatic degradation, facilitate uptake by antigen-presenting cells, and induce strong T-cell responses thus improve the ability of T-cells to kill tumor cells and offer excellent potential in cancer immunotherapy such as in lung cancer. Use of Clustered Regularly Interspaced Short Palindromic Repeats (CRISPR) technology includes various applications such as precise integration, multi-gene editing, and genome-wide functional regulation. Additionally, CRISPR screening using large-scale guide RNA (gRNA) genetic perturbation provides an approach to understand mechanisms underlying anti-cancer efficacy of Chimeric Antigen Receptor (CAR)-T-cell, to modify CAR T-cells and identify new targets (Zhang et al., 2025). Nanocarrier-based co-delivery of Immunotherapeutic Agents for targeting ovarian cancer includes organic (liposomes, Polymeric nanoparticles, dendrimers), inorganic (gold nanoparticles, mesoporous silica nanoparticles, and metal-organic frameworks), and hybrid (polymer-drug conjugates combined with gene vectors, polymer-lipid nanoparticles) nanocarrier systems. Clinical trial data show that such systems can reprogram the myeloid cells in ovarian cancer (Jiao et al., 2026). Co-delivery strategies combine chemotherapy with checkpoint inhibitors, cytokines with adjuvants, and gene therapies with conventional drugs. Nanocarrier-based co-delivery synergistic therapy enhances antitumor immunity by relieving immune suppression, increasing antigen expression, and promoting T-cell infiltration, along with local immune activation with reduced systemic toxicity.

## 5 Biological fate of CBb delivery vectors

Zhao et al. (2024), review investigated the intracellular and subcellular fate of various FDA-approved drug delivery carriers, providing valuable insights into their biological behavior and operational mechanisms *in vivo*. The *in vivo* fate of these carriers encompasses several key aspects, including targeting specificity, biodistribution, circulation time, interactions with cell membranes, intracellular trafficking, endosomal escape, biodegradation, metabolism, and excretion. Following administration, whether by intravenous injection or other delivery routes, drug carriers face various challenges. For intravenously administered carriers, the first challenge is to survive the dynamic blood circulation while avoiding rapid clearance by the immune system before reaching the target tissue and interacting with cells within the tissue. At the subcellular levels, they regulate the therapeutic cargo release, degradation and metabolism, and elimination from the body.

Targeted drug delivery carriers accumulate in specific tissues and then release their therapeutic cargo through different mechanisms: (1) Passive targeting, which relies the enhanced permeability and retention (EPR) effect, allowing nanoparticles (<200 nm) to preferentially accumulate in tumors because of their leaky vasculature and poor lymphatic drainage; (2) Active targeting, which uses ligands such as folic acid, transferrin, peptides, antibodies, aptamers, or oligosaccharides to bind receptors overexpressed on diseased cells, promoting delivery specificity; and (3) Physicochemical targeting, in which stimuli-responsive carriers release their cargo in response to external triggers (e.g., light, heat, ultrasound, or magnetic fields) or disease-specific conditions such as acidic pH or enzyme overexpression, enabling site-specific and controlled drug release.

### 5.1 Bloodstream

Upon entering the bloodstream, drug delivery carriers rapidly adsorb plasma proteins, including serum albumin, lipoproteins, complement proteins, and immunoglobulins, forming a protein corona on their surface (Zhao et al., 2024). This corona promotes recognition by phagocytic cells, leading to accelerated clearance from the circulation and influencing the carrier’s stability, biodistribution, and overall *in vivo* fate. In addition, circulation time is affected by the carrier’s physicochemical properties, particularly its shape.

### 5.2 Drug release

Targeted drug delivery carriers are designed to accumulate in specific tissues and organs after administration. Once they reach the target site of lesions, the subsequent challenge is the controlled release of their therapeutic cargo. Drug release mechanisms vary among carrier systems (Zhao et al., 2024). Some carriers release drugs directly into the extracellular environment at the diseased site, whereas others interact with the cell membrane to deliver their cargo into the cytoplasm without being internalized. In contrast, certain carriers are taken up by cells through specific endocytic pathways and undergo intracellular trafficking to subcellular compartments. During this process, some carriers are degraded, whereas others evade degradation.

Drug delivery carriers interact with cell membranes, to release intracellularly their loaded drug, through two principal mechanisms: (1) endocytosis and (2) membrane fusion. Endocytosis involves the internalization of extracellular materials through plasma membrane deformation, allowing carriers to enter cells. Internalized carriers are typically trafficked to endosomes and lysosomes, where they may undergo degradation unless they possess mechanisms for endosomal escape. In contrast, membrane fusion enables the carrier membrane to directly merge with the cell membrane, releasing the therapeutic cargo into the cytoplasm without cellular internalization. As a result, drugs delivered by membrane fusion bypass the endosomal–lysosomal pathway and avoid intracellular degradation.

Some drug delivery carriers release their therapeutic payload extracellularly upon reaching the target site, such as neutral liposomes and certain physicochemical (stimuli-responsive) carriers. Neutral liposomes exhibit minimal interaction with cell membranes, and therefore their drug release primarily takes place extracellularly. Similarly, physicochemical targeting carriers respond to external or local stimuli at the disease site, triggering structural changes that lead to drug release. In the tumor microenvironment, for example, pH typically ranges from approximately 6.8–7.0 and can decrease to around 5.7 in some cases (Zhao et al., 2024). pH-sensitive carriers exploit these conditions: when they reach the tumor site, their lipids, functional groups, or inorganic components undergo physicochemical changes that destabilize the carrier structure and enhance drug release at the target site.

### 5.3 Degradation and safety

Following drug release step, it is essential to consider degradation and safety of drug delivery biomaterials.

After nanoparticles (NPs) release their therapeutic cargo, surface-associated biomolecules can be degraded by lysosomal enzymes such as cathepsin L (Zhao et al., 2024). Subsequently, NPs of different compositions undergo distinct degradation pathways. For instance, polymer-based NPs are primarily metabolized through the degradation of their polymer backbones, depending on material properties. In general, particles smaller than 5 nm can be rapidly cleared via renal excretion, whereas larger particles and their metabolites are predominantly eliminated through hepatic processing and biliary excretion. However, if NPs escape from lysosomes, they may induce lysosomal disruption, oxidative stress, organelle dysfunction, and the generation of reactive oxygen species (ROS) which can damage cellular components. As a result, despite their utility, some NPs may exhibit low toxicity, including inflammatory responses, oxidative stress, apoptosis, and potential genotoxic and reproductive effects, leading to damage at molecular and genetic levels.

The degradation of extracellular vesicles (EVs), whether derived from eukaryotic cells or prokaryotic microorganisms, is governed by their lipid composition, membrane architecture, and surrounding environmental conditions. Both cell-derived and microbial EVs undergo degradation through several well-defined biochemical and structural mechanisms. Enzymatic degradation is a major pathway, involving lipases, proteases, and nucleases that break down EVs. For example, sphingomyelinase (SMase) specifically degrades sphingomyelin-rich EVs, destabilizing sphingomyelin-containing EVs and releasing their encapsulated biomolecular cargo, which can subsequently serve as a nutrient source for bacteria during nutrient-limited conditions (Laimer-Digruber et al., 2026). Autophagy–lysosomal degradation represents another important mechanism for eukaryotic EVs. Following cellular uptake, exosomes and engineered EVs are engulfed by target cells and ultimately delivered to lysosomes via autophagy, where they are degraded (Zheng et al., 2019). In addition, targeted protein degradation (TPD) has emerged as a novel strategy for selectively eliminating EVs. This approach utilizes the expression of specific transmembrane proteins to promote localized extracellular degradation of specific EVs (Tong et al., 2025).

Concerning microalgae cells, once they reach their target site, such as tumors, the lungs, or the gastrointestinal tract, they are eliminated through natural biological degradation pathways. Lysosomal degradation by macrophages is a primary mechanism following systemic or localized administration. For example, after intratracheal delivery of algae-based microrobots, innate immune cells recognize the microalgae after completing their therapeutic function. Macrophages engulf the cells via phagocytosis and traffic them to acidic lysosomes, where intracellular hydrolases degrade algal proteins, lipids, and carbohydrates into biocompatible metabolites (Wang et al., 2025). In oral drug delivery applications, microalgal biomass can also serve as a prebiotic substrate. Enzymes produced by the gut microbiota, particularly polysaccharide-degrading enzymes, ferment and digest algal cell wall polysaccharides in the large intestine, facilitating their safe metabolism and elimination (Wang et al., 2025). For silica-containing marine-derived microalgae, such as diatoms, the porous biosilica framework undergoes gradual dissolution into non-toxic orthosilicic acid subsequently excreted by the kidneys (Kang et al., 2024).

Another category of cells; red blood cells (RBCs), under normal physiological conditions, do not leave the bloodstream and enter surrounding tissues, except in specialized sites such as the hepatic sinusoids and the splenic interstitium, where the circulation is relatively open. These regions serve as the primary locations for RBC production and removal and are components of the reticuloendothelial system (RES). Macrophages within the spleen and liver efficiently recognize and phagocytose senescent, damaged, or structurally altered RBCs, resulting in their degradation within lysosomes (Muzykantov, 2010).

## 6 Challenges for CBb vectors

The journey to develop CBb drug delivery systems for cancer treatment is fraught with several obstacles, ranging from technical and regulatory challenges to scalability issues and existing knowledge gaps. Each of these must be addressed to harness the full potential of these innovative therapies.

Although various microorganisms such as bacteria, fungi, and microalgae have shown promising capabilities in drug delivery, optimizing these systems remains a complex task. For example, polymeric micelles have demonstrated notable encapsulation efficiency and drug loading capacity, with some values reaching 33.64% and 48.90%, respectively (Mahani et al., 2023). However, achieving consistent results across different microbial platforms presents difficulties due to the variability in microbial growth conditions and metabolic states. Moreover, targeting cancer cells using engineered microorganisms, such as those designed to express specific ligands, demands comprehensive characterization and validation to ensure both efficacy and safety (Pandey and Pandey, 2024).

Regulatory challenges also loom large in the path of clinical translation of microorganism-based drug delivery systems. The therapeutic application of live microorganisms raises significant concerns about safety, potential pathogenicity, and immunogenicity. Regulatory bodies may require extensive preclinical and clinical data to thoroughly assess the risks associated with these novel systems, potentially prolonging the approval process and inflating development costs. Additionally, the regulatory landscape for microbial therapies is still evolving, with a lack of standardized guidelines complicating the journey to market.

Scalability poses another significant challenge. While laboratory-scale experiments have yielded promising results, scaling these findings to a commercially viable production level is a substantial hurdle. For instance, the production of microalgae-based vectors. Despite their biocompatibility and capacity to enhance drug loading efficiency (Huang et al., 2024), factors such as growth rates, biomass yield, purity, pyrogenicity, and extraction processes can limit production. Optimizing cultivation conditions and refining downstream processing techniques are essential to make large-scale production feasible.

Furthermore, there are still knowledge gaps regarding the interactions between microorganisms and the tumor microenvironment. Although studies have indicated that microalgae can effectively deliver anticancer drugs and boost chemotherapy efficiency (Wang et al., 2019), further research is needed to clarify the mechanisms driving these interactions and to pinpoint potential barriers to drug delivery within the tumor context.

In summary, while CBb drug delivery vectors, particularly microalgae, show considerable promise in cancer treatment, overcoming these challenges is essential for successful implementation.

## 7 Clinical translation of CBb vectors

The clinical translation of microorganism-based drug delivery vectors for cancer treatment has made notable progress, yet several obstacles persist that demand further investigation and development. Various platforms, like micelles, magnetosomes, red blood cells, and microbial systems, have shown differing levels of efficacy in preclinical studies. However, bridging the gap between these promising results and clinical application presents substantial challenges.

Take polymeric micelles, for instance. Dap-DOX micelles have demonstrated increased cytotoxicity compared to free DOX, with selective tumor site accumulation due to the enhanced permeability and retention (EPR) effect (Guo et al., 2021). Nonetheless, their clinical use is limited by issues related to their stability in biological settings and the reproducibility of their synthesis. Thus, our research should aim to optimize formulation stability and conduct detailed pharmacokinetic studies to better understand their *in vivo* behavior.

Similarly, magnetosomes, especially drug-loaded bacterial magnetosomes (DBMs), have shown significant tumor suppression and reduced cardiac toxicity compared to traditional DOX treatments (Sun et al., 2007). Despite these benefits, their clinical translation is hampered by the challenges of large-scale production and the need for precise magnetic targeting. Future endeavors should focus on developing scalable production techniques and integrating external magnetic fields to enhance targeting accuracy.

RBC-based drug delivery systems have effectively loaded and delivered DOX to cancerous tissues, showing superior anticancer activity compared to free DOX (Wu et al., 2021). However, their clinical application is complicated by the necessity of autologous sourcing and potential immunogenic reactions. Developing universal donor RBCs or synthetic alternatives could potentially overcome these hurdles, enhancing their clinical applicability.

Microalgae present a particularly promising platform due to their excellent biocompatibility and enhanced drug loading efficiency (Huang et al., 2024). Their capability to produce oxygen under laser irradiation to mitigate tumor hypoxia further highlights their potential for improving chemotherapy outcomes (Wang et al., 2019). Nevertheless, the clinical application of microalgae-based systems remains in its early stages, requiring significant research to establish safety profiles, optimize drug loading methods, and assess efficacy in human trials.

Although microalgae such as Spirulina have traditionally been investigated as nutritional supplements (Wang et al., 2026), recent studies have expanded their applications to include living photosynthetic scaffolds and targeted drug delivery platforms. In a first-in-human clinical trial, Obaíd et al. (2021), demonstrated the safety and feasibility of implanting microalgae-based photosynthetic scaffolds for tissue oxygenation. Treatment of eight patients with full-thickness skin wounds resulted in no local or systemic immune reactions during a 90-day follow-up period and promoted complete tissue regeneration. These results support the clinical potential of photosynthetic biomaterials and their future use in tissue engineering and regenerative medicine.

To date, magnetic microalgae have not entered clinical trials for drug delivery and remain under preclinical investigation, with research primarily limited to laboratory and animal models (Liu Y. et al., 2025). Before these systems can be translated into clinical practice, several key challenges must be overcome, including the optimization of microalgal carriers, improvement of drug loading efficiency, integration of multimodal therapeutic approaches, and thorough safety assessment in large animal studies. Owing to their renewable nature and distinctive biological characteristics, microalgae represent a promising platform for future drug delivery, diagnostic, and therapeutic applications.

Beyond these specific challenges, a universal hurdle for all microorganism-based platforms is the regulatory landscape, which tends to be especially stringent for biologically derived products. Collaborative efforts among researchers, clinicians, and regulatory bodies will be crucial to navigate these complexities and facilitate the transition from research to clinical practice.

## 8 Conclusion

Our investigation into CBb drug delivery systems for cancer therapy reveals exciting potential, suggesting these vectors could significantly improve treatment effectiveness while reducing systemic toxicity. Notably, various platforms such as micelles, RBC, bacterial OMVs, and microalgae show promising improvements in targeted drug delivery and controlled release. For example, Dap-DOX micelles display enhanced cytotoxicity over free DOX and tend to accumulate at tumor sites via the enhanced permeability and retention (EPR) effect, indicating a potential advantage for localized treatment (Guo et al., 2021). Similarly, red blood cells used as carriers have effectively targeted cancer cells, with Dox-gluRDVs showing superior anticancer activity both *in vitro* and *in vivo* (Wu et al., 2021).

In addition, engineered microorganisms like magnetosomes and microalgae offer innovative solutions to the challenges of traditional drug delivery methods. Magnetosomes, in particular, have achieved an impressive tumor suppression rate of 86.8%, while also demonstrating reduced cardiac toxicity compared to conventional DOX treatments (Sun et al., 2007). Microalgae also stand out due to their excellent biocompatibility and their capacity to enhance drug loading efficiency, which is particularly beneficial in acidic tumor microenvironments (Delasoie et al., 2020; Bolaños-Martínez et al., 2022; Picciotto et al., 2022; An et al., 2024; Huang et al., 2024; Garaeva et al., 2025). These findings emphasize the flexibility and promise of microorganism-based systems in potentially overcoming the limitations of current therapeutic modalities.

Bacteria-based drug delivery systems offer innovative approaches for targeted cancer therapy, though they come with challenges related to safety, immunogenicity, and production scalability. The potential for engineered microorganisms, including bacteria, to boost anti-cancer efficacy while minimizing toxicity is encouraging and aligns with the broader investigation into microbial-based vectors, such as microalgae, which might provide additional advantages in drug delivery (Walker et al., 2019; Schmidt et al., 2020; Bruno et al., 2025).

Nevertheless, several challenges and uncertainties persist. The scalability of these biotechnological approaches for clinical use has not yet been fully realized. Furthermore, although *in vitro* and *in vivo* results are encouraging, additional studies are necessary to assess long-term safety, efficacy, and the potential for immune responses in human subjects. More comprehensive research is also required to refine the engineering of microorganisms to express specific ligands for targeted delivery, ensuring precision and minimizing off-target effects (Wang et al., 2019; Adamo et al., 2021; Bolaños-Martínez et al., 2022; Xu et al., 2022; Mahani et al., 2023; Inukai et al., 2024; Liang et al., 2024; Pandey and Pandey, 2024).

While existing evidence supports the promise of CBb drug delivery vectors in cancer treatment, further research is crucial to address current challenges and validate these systems in clinical settings. Continued exploration in this area holds the potential to develop innovative therapeutic strategies leveraging the unique properties of microorganisms, ultimately improving outcomes for cancer patients. Integrating these novel platforms into standard treatment regimens could mark a significant milestone in the ongoing fight against cancer.
